# Woodland Dynamics at the Northern Range Periphery: A Challenge for Protected Area Management in a Changing World

**DOI:** 10.1371/journal.pone.0070454

**Published:** 2013-07-29

**Authors:** Scott L. Powell, Andrew J. Hansen, Thomas J. Rodhouse, Lisa K. Garrett, Julio L. Betancourt, Gordon H. Dicus, Meghan K. Lonneker

**Affiliations:** 1 Department of Land Resources and Environmental Sciences, Montana State University, Bozeman, Montana, United States of America; 2 Department of Ecology, Montana State University, Bozeman, Montana, United States of America; 3 National Park Service, Upper Columbia Basin Network, Bend, Oregon, United States of America; 4 National Park Service, Southeast Region, Atlanta, Georgia, United States of America; 5 U.S. Geological Survey, National Research Program, Reston, Virginia, United States of America; 6 National Park Service, Upper Columbia Basin Network, Moscow, Idaho, United States of America; 7 National Park Service, Upper Columbia Basin Network, Moscow, Idaho, United States of America; University of Sydney, Australia

## Abstract

Managers of protected natural areas increasingly are confronted with novel ecological conditions and conflicting objectives to preserve the past while fostering resilience for an uncertain future. This dilemma may be pronounced at range peripheries where rates of change are accelerated and ongoing invasions often are perceived as threats to local ecosystems. We provide an example from City of Rocks National Reserve (CIRO) in southern Idaho, positioned at the northern range periphery of pinyon-juniper (P-J) woodland. Reserve managers are concerned about P-J woodland encroachment into adjacent sagebrush steppe, but the rates and biophysical variability of encroachment are not well documented and management options are not well understood. We quantified the rate and extent of woodland change between 1950 and 2009 based on a random sample of aerial photo interpretation plots distributed across biophysical gradients. Our study revealed that woodland cover remained at approximately 20% of the study area over the 59-year period. In the absence of disturbance, P-J woodlands exhibited the highest rate of increase among vegetation types at 0.37% yr^−1^. Overall, late-successional P-J stands increased in area by over 100% through the process of densification (infilling). However, wildfires during the period resulted in a net decrease of woody evergreen vegetation, particularly among early and mid-successional P-J stands. Elevated wildfire risk associated with expanding novel annual grasslands and drought is likely to continue to be a fundamental driver of change in CIRO woodlands. Because P-J woodlands contribute to regional biodiversity and may contract at trailing edges with global warming, CIRO may become important to P-J woodland conservation in the future. Our study provides a widely applicable toolset for assessing woodland ecotone dynamics that can help managers reconcile the competing demands to maintain historical fidelity and contribute meaningfully to the U.S. protected area network in a future with novel, no-analog ecosystems.

## Introduction

Accelerated climate change presents daunting challenges to managers of protected natural areas at poleward and leading edges of species distributions [1], [2]. These areas are inherently dynamic and are likely to experience some of the most pronounced changes in community composition during an era of global change [3], [4], [5]. Where dominant native species expand into new terrain, such “natural invasions” could reduce and even eliminate other species and communities, a perceived negative outcome. Presumably, these changes could be reversed by appropriate management actions. However, invading native species may also provide beneficial habitat value and may be vulnerable to contraction and decline in other parts of their ranges. Native invaders, as peripheral populations, may harbor important genetic diversity and increased capacity for resilience to climate change and thereby increase the conservation value of protected areas at the range periphery [4], [6], [7]. Furthermore, efforts to slow or remove invaders may themselves be counterproductive. For example, controlled fires used to remove or thin native trees and shrubs can inadvertently facilitate invasions by non-native weeds [8], [9], [10]. This issue is further complicated in protected areas where other values and goals, such as the preservation of iconic landscapes, also influence the decision-making process.

Historically, the management of protected areas has been founded upon the assumption that ecosystem development is inherently linear and that ecosystems develop predictably along a single successional pathway [10], [11], [12]. But the accelerating rates of biological invasions and climate change, and a greater appreciation for the long-lasting legacies of past human land use, have heightened awareness for the inherently dynamic and non-linear nature of ecological change [9], [10], [13]. As plant and animal communities disassemble and reassemble in unique ways, protected area managers are being forced to change their perceptions of what is “natural” or “desirable” in the ecosystems they manage [2]. These so-called “novel ecosystems” [14], [15], including those newly dominated by formerly subdominant or peripheral native species, will be increasingly unfamiliar to protected area managers. Confronted with novel ecosystems under their stewardship, managers will have to increasingly consider external ecological phenomena and trends that can impact protected areas, and embrace an unprecedented degree of coordination across other reserves in the region [10], [16]. Furthermore, protected area managers will need to gain a better understanding of the local dynamics and history of novel ecosystem development [2], [17].

**Figure 1:**
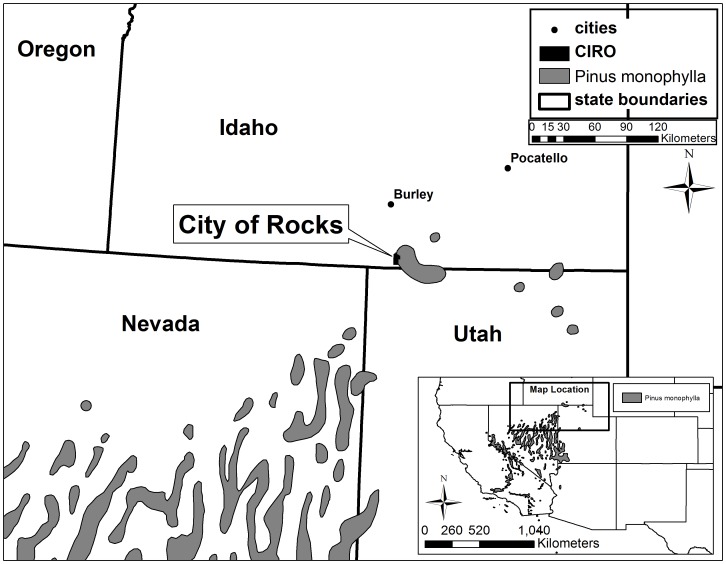
Map of the location of City of Rocks National Reserve, with respect to the range of *Pinus monophylla* (from [60]).

**Figure 2:**
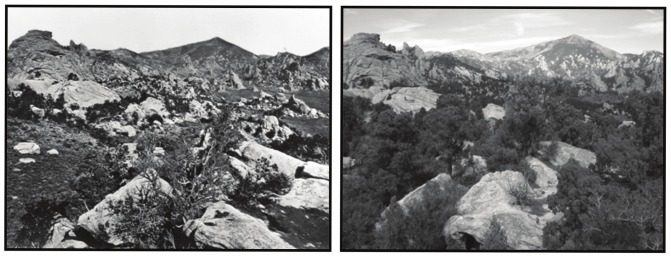
Repeat photographs from CIRO from 1868 (left) and 2005 (right) showing increase in woody vegetation (from [20]).

To motivate this discussion and to provide an accessible toolset for managers and supporting conservation scientists that can be used to quickly gain this better understanding of local woodland dynamics, we describe a case study of change in the pinyon-juniper (P-J) woodlands of City of Rocks National Reserve, in southern Idaho, USA. City of Rocks (CIRO) is located at the northern terminus of the distribution of *Pinus monophylla* and *Juniperus osteosperma* (Fig. 1), as well as at the northern range periphery of several co-occurring P-J woodland obligate rodents and birds [18]. Local woodlands contribute meaningfully to regional biodiversity and also to the striking “city of rocks” iconic landscape enshrouding many of the huge granite monoliths that give the reserve its name. However, in CIRO, repeat photography shows both tree densification (infilling) within historic P-J woodland stands and P-J woodland encroachment out into sagebrush steppe [19], [20] (Fig. 2). This phenomenon of P-J woodland expansion is widespread throughout much of the western American sagebrush biome [21], [22] and is widely seen as a serious threat to the ecological integrity of sagebrush steppe [23]. P-J woodland removal through prescribed burning and mechanical methods is recommended practice to protect sagebrush steppe and to promote forage production for domestic livestock and wildlife [24], [25]. CIRO reserve managers are faced with deciding whether and where to take these same actions. However, the unique historical and ecological considerations associated with CIRO’s protected-area status and its position along the northern range periphery of the P-J woodland adds to the complexity of the decision-making environment. Without specific understanding of how P-J woodlands in the reserve have developed and changed, decision-making is hampered further.

To assist CIRO managers with their decision-making, we assessed P-J woodland change in the reserve using simple, repeatable aerial photo interpretation over the period from 1950 to 2009. Specifically, we quantified rates of change in the spatial extent of P-J woodlands as well as the variability in the rates of change along biophysical gradients in CIRO. Our method has been applied previously to high-elevation conifer forests [26], but for this study we were motivated to demonstrate its utility in a protected area setting. We recognized that protected area managers in CIRO and in other reserves along the northern P-J woodland range periphery, including John Day Fossil Beds National Monument and Big Horn Canyon National Recreation Area, are faced with difficult decisions about whether and how to slow encroachment of these woody native species that are seen as a threat to ecological integrity but that are experiencing die-off in other parts of their ranges [27]. Our study describes how an assessment of change in a particular protected area, CIRO, yielded surprising and important insights relevant to current decision-making. The methods employed are accessible and easily reproduced for use in other protected areas and land management settings. We provide these as supplemental standard operating procedures in Appendix S2.

## Methods

### Study Area

The 5,830 ha City of Rocks National Reserve in southern Idaho, USA lies within the Albion Mountains, an isolated massif in the eastern Snake River Plain (Fig. 1). Elevation in CIRO ranges from 1,646 m in the southern portion of the reserve to 2,706 m on the tallest summit in the northern portion. Current vegetation includes exotic annual grassland and sagebrush (*Artemisia spp.*) steppe at lower elevations, pinyon-juniper (*P. monophylla*-*Juniperus spp.*), mountain mahogany (*Cercocarpus ledifolius*), and aspen (*Populus tremuloides*) woodlands at mid-elevations, and Douglas-fir (*Psuedotsuga menziezii*), lodgepole pine (*Pinus contorta*), and subalpine fir (*Abies lasiocarpa*) forest at the upper elevations [28]. Fourteen documented fires occurred in CIRO between 1926 and 2005, with the largest in 2000 burning over 7,000 ha of P-J woodland and sagebrush steppe in the southern portion of the reserve [29] (Appendix S1). The non-native invasive annual grass cheatgrass (*Bromus tectorum*) has invaded much of the recently burned area [30]. Crested wheatgrass, a non-native perennial bunchgrass widely planted as livestock forage in the western U.S. is also abundant in many of the low-elevation, flatter portions of the reserve. Our study area encompassed the entirety of CIRO along with a small peripheral buffer of approximately 1–4 km. We selected the boundary for the study buffer based upon the availability and coverage of digital aerial photos and ancillary GIS data layers (Appendix S2) (Fig. 1).

### Sampling Design

We stratified the study area based upon three key biophysical gradients in the reserve: vegetation (type and density), elevation, and solar radiation (slope and aspect). To do so, we compiled a suite of biophysical GIS data layers for CIRO, including a 2010 reserve vegetation map [28], digital elevation model (DEM), and a solar radiation model derived from the DEM, to capture the range of ecological variation across the reserve. We stratified the DEM into three classes: low elevation (1,672 m –1,974 m), mid elevation (1,947 m –2,177 m), and high elevation (2,177 m –2,691 m). We calculated solar radiation from the DEM using the Area Solar Radiation tool in ArcMap (ESRI version 10.0) and stratified it into three classes: low radiation, mid radiation, and high radiation. We reclassified the 45 vegetation map classes from Erixson and Cogan [28] into four physiognomic classes (evergreen woodland, herbaceous, shrubland, deciduous woodland), and an unvegetated class that contained rock, water, and other land cover. For each of the four vegetation classes, we further separated them into high (>60%) and low (≤60%) density classes based on the percent cover of the upper stratum layer [28]. This resulted in eight total vegetation classes. We then combined the eight vegetation classes, three elevation classes, and three solar radiation classes into a single grid layer with 72 possible strata (68 realized). From this layer, we generated a stratified random sample of 340 sample plots (5 plots per stratum), each of dimension 100 m×100 m (1 ha). Additional details for developing the stratified grid layer are provided in Appendix S2.

### Aerial Photo Characteristics and Interpretation

We used digital aerial photos from 1950, 1990, and 2009 for change detection. The 1950 and 1990 photos were archived in a National Park Service collection. The 1950 photos were digitized from black and white aerial print photos at 1 m resolution. The 1990 photos also were digitized from color aerial print photos at 1 m resolution. The 2009 photos were obtained from the U.S. Department of Agriculture National Agriculture Imagery Program (NAIP) as color 1 m imagery (Available online at: http://www.fsa.usda.gov/FSA). Within each of the 340 sample plots, we generated a random sample of 10 points to guide aerial photo interpretation based on the point-intercept method which relies on a tally of intersections between points and vegetation types [26] (Appendix S2). Within each plot, we quantified the percent composition of evergreen woodland, deciduous woodland, herbaceous/shrub, or other (e.g. rock) in 10% increments. We further classified evergreen vegetation as either needleleaf (conifer) or broadleaf (e.g. mountain mahogany), and then based on descriptions and photographic examples from Miller et al. [31] (Appendix S2), determined which of the three transitional phases of P-J woodland succession the plot most closely resembled, from phase I (initial woodland encroachment) to phase III (mature, closed-canopy woodland). Finally, we assessed overt signs of recent disturbance due to fire, insects, or harvest activity. Table 1 provides a summary of this hierarchical scheme. The 1950 photographs were black and white and of inferior quality to the color 1990 and 2009 photographs. Therefore, we conservatively quantified the percent composition of broad vegetation classes in coarse increments of 10% to mitigate potential errors associated with shadows and misregistration.

**Table 1:** The hierarchical scheme used to classify 1 ha. aerial photograph plots for woodland change detection in City of Rocks National Reserve.

Level 1	Level 2	Level 3	Level 4
% Composition	Type	Transitional Phase	Disturbance
% Evergreen	Needleleaf	Phase I	Fire
	Broadleaf	Phase II	Insect
		Phase III	Harvest
			Other
% Other	Bare		
	Rock		
	Agriculture		
	Water		
% Herbaceous/Shrub		Phase 0	
% Deciduous			

To quantify the plot-level woodland vegetation rate of change, we calculated the change in percent evergreen composition between time periods and divided by the number of years between observations. For assessment of variation in rates of change across the full sample of plots, we estimated pairwise differences among all of the biophysical sampling strata using the Bonferonni alpha correction procedure, and reported statistically significant differences only when 95% family-wise confidence intervals did not include zero. This approach was chosen to protect against inflated Type I errors greater than the nominal 0.05% rate given the number of pairwise comparisons required (Appendix S2).

To estimate the spatial extent of changes in conifer, sagebrush steppe, and grassland communities, we reclassified the 2010 CIRO vegetation map [28] and the sample plots into four broad classes: 1) sagebrush/shrub/herbaceous/pinyon-juniper, 2) mahogany/deciduous, 3) other conifer, and 4) other. We grouped sagebrush, shrub, herbaceous, and pinyon-juniper map classes together in order to focus on ecotonal dynamics between sagebrush steppe and pinyon-juniper woodland communities. There were 244 sample plots that overlapped this sagebrush/shrub/herbaceous/pinyon-juniper class, and we assigned each of these plots to a woodland succession phase at each time period. All 244 plots, therefore, fell along a continuum from phase 0 of woodland succession (e.g. no tree cover) to phase III (100% pinyon-juniper cover). The proportion of plots in each of the phases was then multiplied by the aerial extent of phases based on the 2010 vegetation map to determine the transitional phase areas for each observation period. Phase transitions were then tallied between time periods to determine the relative magnitudes and directions of change. Additional details are provided in Appendix S2.

## Results

### Vegetation change between 1950 and 2009

**Figure 3:**
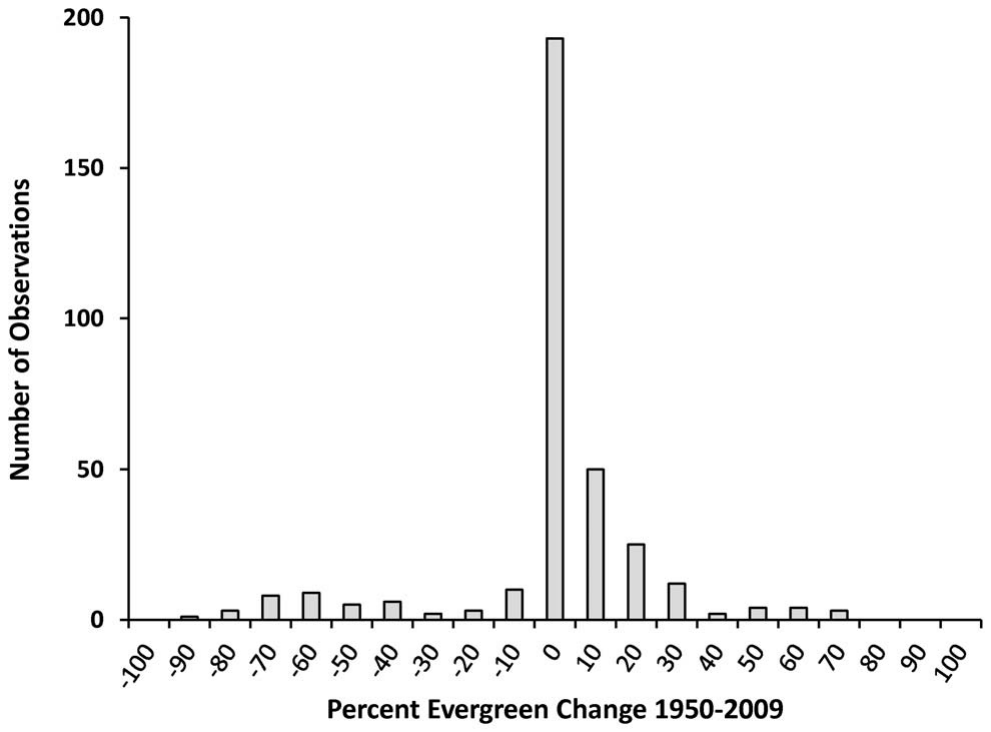
Distribution of changes in percent evergreen vegetation cover between 1950 and 2009 for the 340 sample plots.

The observed plot-level changes (*n* = 340) in woodland cover between 1950 and 2009 were extremely heterogeneous, ranging from 90% loss of woodland cover due to disturbance to a gain of 70% woodland cover due to encroachment and densification (Fig. 3). The majority of plots (57%) exhibited no change in woodland cover between 1950 and 2009 (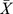 = −0.15, SD = 23.87). Overall, average woodland cover remained at approximately 20% between 1950 and 2009, increasing from 20% in 1950 to 26% in 1990, and then decreasing to 20% in 2009.

**Figure 4:**
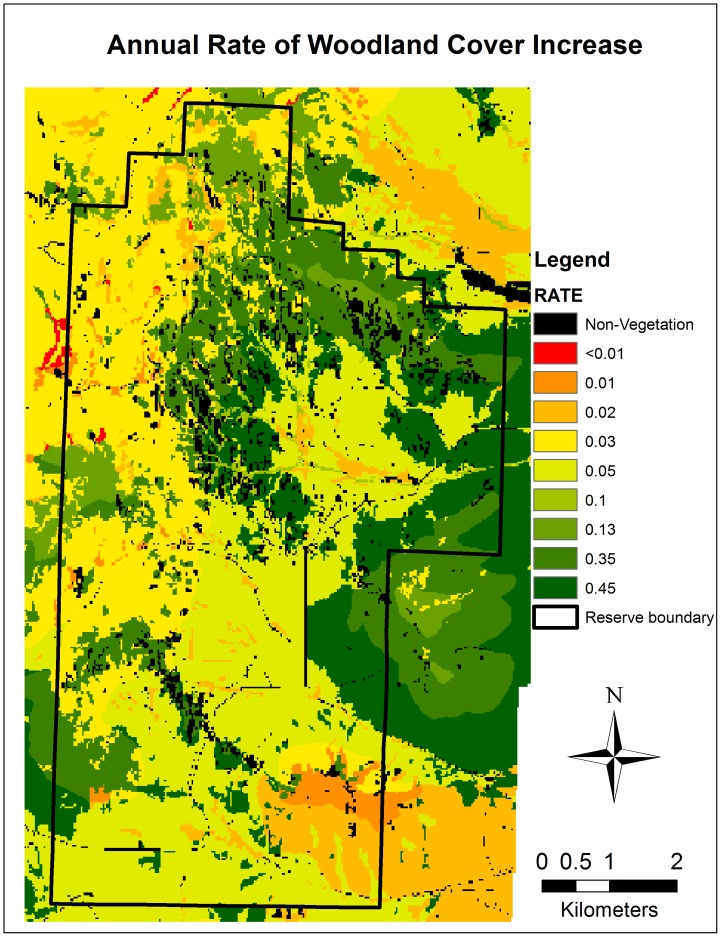
Annual rate of woodland cover increase at City of Rocks National Reserve between 1950 and 2009 by vegetation and elevation gradients. Rates were spatially extrapolated according to estimated rates of change by vegetation and elevation strata (Table 2).

Rates of intrinsic woodland cover increase, measured from plots not disturbed by fire or other forces during the 60-year study period (*n* = 284), varied significantly by vegetation type and biophysical setting (Table 2 and Fig. 4). Woodland densification was significantly more rapid (0.30% yr^−1^) than woodland encroachment into deciduous, herbaceous, or shrub vegetation (Table 2). However, the rates of woodland cover increase did not differ significantly among density classes (Table 2). Rates of cover increase in pinyon-juniper woodland plots were significantly higher (0.37% yr^−1^) than in all other vegetation types except for other conifer (0.22% yr^−1^; Table 2). Rates of cover increase in mahogany-dominated plots were also high (0.13% yr^−1^) but the differences were not statistically significant from other vegetation types. Low elevation areas (0.20% yr^−1^) exhibited significantly higher rates of cover increase than mid elevation areas (0.11% yr^−1^) and high elevation areas (0.06% yr^−1^). There was no significant variation in rates of change along the solar radiation gradient, and there was no evidence for a meaningful interaction between elevation and solar radiation (Table 2).

**Table 2:** Rates (% per year) of intrinsic woodland cover increase by biophysical and vegetation gradients, including Bonferonni corrected 95% confidence intervals (Lower CI and Upper CI).

Variable	Category	Rate	SE	LowerCI	UpperCI	Diff
elevation	high	0.06	0.02	0.01	0.11	a
	mid	0.11	0.02	0.06	0.16	ab
	low	0.20	0.02	0.15	0.26	c
solar radiation	high	0.15	0.02	0.09	0.20	a
	mid	0.10	0.02	0.04	0.15	a
	low	0.11	0.02	0.05	0.17	a
elevation/solar radiation	high/high	0.09	0.04	−0.01	0.19	a
	high/mid	0.04	0.04	−0.06	0.14	a
	high/low	0.04	0.04	−0.07	0.14	a
	mid/high	0.15	0.04	0.05	0.25	a
	mid/mid	0.07	0.04	−0.03	0.17	a
	mid/low	0.11	0.04	−0.02	0.23	a
	low/high	0.22	0.04	0.10	0.33	a
	low/mid	0.19	0.04	0.08	0.29	a
	low/low	0.21	0.04	0.09	0.34	a
vegetation class	evergreen	0.30	0.02	0.26	0.35	a
	deciduous	0.04	0.02	−0.01	0.09	b
	herbaceous	0.01	0.03	−0.07	0.09	b
	shrub	0.04	0.02	−0.01	0.08	b
vegetation type	sagebrush	0.03	0.02	−0.02	0.08	a
	mahogany	0.13	0.03	0.04	0.22	a
	deciduous	0.03	0.02	−0.04	0.09	a
	herbaceous	0.01	0.03	−0.09	0.10	a
	other conifer	0.22	0.07	0.04	0.40	ab
	pinyon-juniper	0.37	0.02	0.31	0.42	b
	rocky-outcropping	0.04	0.09	−0.20	0.28	a
	shrub	0.02	0.02	−0.13	0.17	a
vegetation/density	evergreen/high	0.34	0.03	0.25	0.42	a
	evergreen/low	0.28	0.03	0.21	0.35	a
	deciduous/high	0.04	0.03	−0.04	0.12	b
	deciduous/low	0.04	0.03	−0.05	0.13	b
	herbaceous/high	0.02	0.06	−0.14	0.18	b
	herbaceous/low	0.01	0.04	−0.10	0.11	b
	shrub/high	0.02	0.03	−0.06	0.09	b
	shrub/low	0.05	0.03	−0.02	0.13	b
vegetation/elevation	evergreen/high	0.13	0.03	0.05	0.09	a
	evergreen/mid	0.35	0.03	0.26	0.45	b
	evergreen/low	0.45	0.03	0.36	0.54	b
	deciduous/high	0.02	0.03	−0.07	0.09	a
	deciduous/mid	0.03	0.03	−0.07	0.12	a
	deciduous/low	0.10	0.04	−0.02	0.22	a
	herbaceous/high	0.00	0.05	−0.14	0.14	a
	herbaceous/mid	0.01	0.05	−0.12	0.15	a
	herbaceous/low	0.02	0.06	−0.15	0.19	a
	shrub/high	0.03	0.03	−0.06	0.12	a
	shrub/mid	0.03	0.03	−0.06	0.11	a
	shrub/low	0.05	0.03	−0.04	0.14	a

Within-variable rates with the same Diff letter do not differ significantly from one another as determined by the Bonferroni pairwise comparison procedure.

### Changes in the Spatial Extent of Vegetation Communities Across CIRO

**Figure 5:**
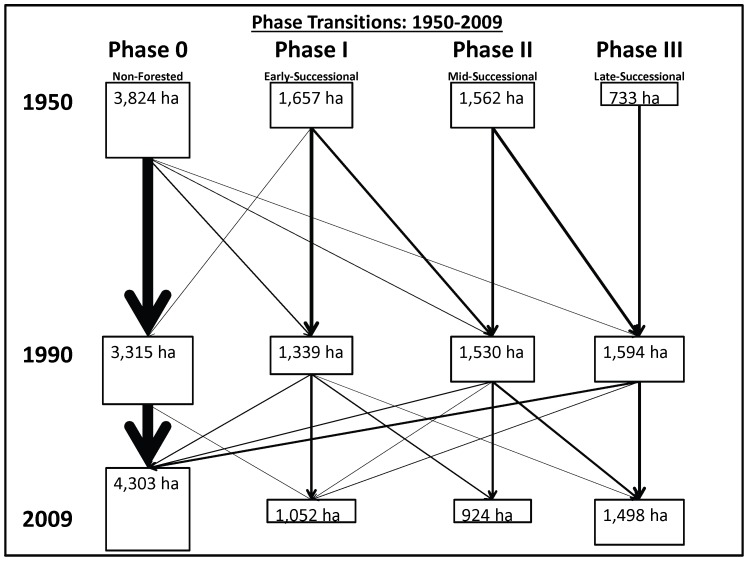
Phase transitions between 1950 and 2009 at CIRO. The sizes of the boxes are proportional to the areal extent and the sizes of the arrows are proportional to the magnitude of the transitions.

Disturbances, mostly fire (85%), were recorded in 16% of the sample plots between 1950 and 2009. All of these plots contained woodland prior to burning, and 77% shifted to invasive annual grasslands following fire. Prior to the extensive fires of 1999 and 2000 in the southern portion of CIRO, woodland encroachment between 1950 and 1990 resulted in a shift of approximately 13% of grasslands and shrublands (phase 0) to woodland (Fig. 5). During that same time period, densification in phase I and phase II stands resulted in a 117% increase in phase III woodland extent across the study area.

Following the fires of 1999 and 2000, the overall extent of grassland and shrubland areas increased across the study area as phase I, II, and III stands reverted to phase 0 (Fig. 5), with 85% of these shifting to invasive annual grasslands. Over the 60-year study period, non-forested areas increased by 13% overall, while phase I and phase II woodland areas decreased by 37% and 41%, respectively. Phase III woodlands increased in area by over 100% over the study period, despite the loss of 7% of phase III plots to fire between 1990 and 2009.

## Discussion

Through straightforward and repeatable aerial photointerpretation methods, we documented the recent development of an increasingly novel ecosystem positioned at the P-J woodland northern range periphery. Based on our study beginning in 1950, CIRO vegetation has been shifting away from a mosaic of open canopy P-J woodlands and sagebrush steppe towards one dominated by exotic annual grasslands in the southern, low-elevation portion of the reserve and closed canopy P-J woodlands in the northern and eastern portions of the reserve. Early- and mid-successional P-J stands have declined in extent and the overall footprint of the P-J woodland in the reserve has not increased. But recent monitoring shows that the area of cheatgrass infestation has increased, particularly in the southern part of the reserve [30], [32]. Stands of sagebrush steppe relatively uninfested by cheatgrass are still common in the northern, higher elevation portions of the reserve [32]. These documented trajectories of change in CIRO have no historic or pre-historic analog.

Documented changes in the extent of P-J woodlands in CIRO are similar to trajectories reported in other areas of the western U.S. Reported extents of P-J woodland cover increase since the late 1800s range from 140% to over 600% [31], [33], [34] and in southern Idaho Sankey and Germino [35] estimated 22–30% encroachment over a 20-yr time period beginning in 1985. Paleoecological studies in the region suggest that the sagebrush steppe/pinyon-juniper ecotone was highly dynamic during the late Pleistocene and Holocene, driven by extreme climatic variability and drought cycles [22], [36], [37], [38], [39]. The northern edge of the range of *P. monophylla* is thought to have reached its current northern range limit in the vicinity of CIRO approximately 2,800 years ago but did not infill across the entire reserve until after 700 years ago [40]. By the time of Euro-American settlement across the western U.S. in the mid-19^th^ century, the density of woodland cover was likely less than 10% of today’s conditions [31]. P-J woodlands expanded rapidly after 1860, with the most rapid rates occurring during 1880 to 1920 in Idaho and between 1900 and 1920 in Utah, Nevada, and Oregon [31]. The series of historic photographs [19], [20] available for CIRO begin in the 1860s and indicate accelerated rates of P-J woodland expansion and densification in the reserve since that time. Cheatgrass, crested wheatgrass, and other exotic species were established by the early 20^th^ century [41], [42], which in concert with changing climate and land use have profoundly altered fire regimes, nutrient cycling, and other ecosystem processes [8], [43].

The relative importance of climate variability, climate change, and land use in P-J dynamics are not well understood and apparently vary across western P-J woodlands [22], [31], [44], [45]. Within the northern Great Basin, including the vicinity of CIRO, Miller et al. [31] concluded that the shift from a relatively limited rate of woodland establishment in the mid-1800s to a substantially increased rate in the early 1900s was likely due to the combination of factors including reduced role of fire, introduction of domestic livestock grazing, and a shift in climate towards cooler and wetter conditions. Soule et al. [45] arrived at similar conclusions from a study of western juniper populations also at the northern range periphery, as did Weisberg et al. [46] in the core of the P-J woodland range in Nevada. Within CIRO, historical photographs indicate that P-J woodland was largely confined to rock outcrops in the mid-1800s (e.g., Figure 2). This is consistent with the hypothesis that fire excluded P-J woodland from deeper valley soils [31].

Our study highlights several profound challenges associated with protected area management decision-making in the context of ecological novelty at the range periphery. In such a complex setting, managers will increasingly be faced with competing paradigms. On the one hand, protected areas are conceptually and in some cases administratively committed to maintaining historical fidelity, guided by concepts such as historic range of variability [2], [12], [47], [48]. On the other hand, protected areas positioned at the range periphery are the most likely to experience rapid environmental changes that shift ecosystems away from historical conditions, in some cases because peripheral, isolated populations can grow faster as a result of escape from obligate herbivores and pathogens that are more common in range cores [37], [49]. The costs and technological know-how required to prevent a-historical change in most cases will exceed capacity, requiring either a change in objectives away from historical fidelity or a much more focused management strategy that utilizes triage and prioritization to guide highly targeted actions [2], [10], [48]. These management challenges are further compounded by the fact that developing novel ecosystems are unlikely to be static [17] and hence will require frequent monitoring and continuously updated management approaches. The conflict is all the more challenging in cases where iconic landscape components that are integral to the identity of a protected area are subject to rapid change. The scenario of Joshua tree (*Yucca brevifolia*) extirpation at Joshua Tree National Park and potential range expansion elsewhere provides a striking example [13], [50]. In the case of CIRO, our study suggests that the conservation of the mid-1800s California Trail viewshed through the reserve, which is dependent on the mosaic of sagebrush steppe and P-J woodland visible in Figure 2 (left panel), will become an increasingly costly management objective given high rates of woodland densification, annual grass invasion, and altered fire frequency and other ecological processes.

An alternative strategy, representing a paradigm shift in protected area management takes a more dynamic and pluralistic view of protected area identity [2], [49]. Such an approach would allow protected area managers to embrace shifting goals and objectives over time as ecological conditions change. For CIRO, this might involve a shift in emphasis toward P-J woodland conservation, in recognition that P-J woodlands at the southern, trailing edge of the range in the southwestern U.S. have actually been experiencing broad scale dieoffs during droughts exacerbated by hotter growing seasons [27]. Rodhouse et al. [18] also showed that P-J woodland stands with old-growth characteristics in CIRO supported regionally unique rodent species which also reach their northern range limit in and around the reserve. A suite of unique bird species associated with P-J woodland habitat also contributes to the elevated regional biodiversity [51], [18]. The increase in CIRO’s late-successional P-J woodland stands in some portions of the reserve since 1950 presumably has facilitated increased abundances of some of these associated species.

Allowing for “identity change” within protected areas of course presents its own set of challenges. It requires institutional flexibility whereby enabling legislation and mission statements are either written with an emphasis on ecological processes rather than on fixed community assemblages or are allowed to be updated periodically. It also requires greater awareness and coordination among the network of protected areas relevant to the system in question. For CIRO to emerge with renewed focus on P-J woodland conservation requires managers to understand that P-J woodlands are in decline elsewhere in the range, and that it is one of the few protected areas along the northern range periphery that may become part of the core range at some point in the future. A more immediate challenge is the tradeoff from the possible loss of sagebrush steppe and associated biodiversity. Accepting a shift toward increasing P-J woodland dominance requires an acceptance of new assemblages of associated flora and fauna. Sagebrush-obligate birds, in particular, are in decline in the region [52] and CIRO currently is home to many of those species as well (CIRO bird species list available online: https://irma.nps.gov/App/Species/Search). Accepting that many such species may be lost from CIRO at some point in the future (but that other new and potentially desirable species will arrive) will challenge the fortitude of reserve managers, and may be simply unacceptable (albeit unavoidable) to some.

Our study and others [30], [32] provide compelling evidence that both the sagebrush steppe and P-J woodland in CIRO are threatened with conversion to annual grassland via wildfire. This represents the most likely pathway for future rapid ecological change in the reserve, and is clearly undesirable when considered from the perspective of maintaining historical fidelity or promoting future biodiversity and resilience to degradation. The positive feedback loop between increased annual grass invasion and accelerated fire frequency [8], [43] will destroy CIRO’s opportunity to maintain healthy sagebrush steppe and P-J woodlands. The southern, low-elevation portion of the reserve has had larger and more frequent fires in recent decades (Appendix S1), and this trend is likely to continue to creep north into higher elevations under predicted scenarios of climate change for the region, which include declining snowpack and aridification [53], [54]. Indeed, intensified and prolonged drought events in the southwestern U.S. have accelerated P-J woodland die-off [27] and, in conjunction with fire, are thought to have shaped P-J woodland dynamics during the Holocene as well [37].

We conclude by considering what kinds of actions CIRO managers should consider in the context of the present discussion to promote biodiversity and ecological resilience into the future. Our study has revealed several important and somewhat surprising insights that might lead to specific actions. First, we determined that encroachment of P-J woodlands out into sagebrush steppe has not been occurring as rapidly as has been feared by CIRO managers, held in check at least in part by recent wildfires. Conversion of steppe and former woodland to novel cheatgrass-dominated steppe via wildfire has been rapid. This suggests that managing tree encroachment into sagebrush steppe per se may be a less immediate concern than managing fire risk, which presents a serious management challenge given the well documented increases in fine fuels, flammability, and associated fire return interval of cheatgrass systems compared to other Great Basin vegetation types [55], [56], [57]. Second, we determined that early- and mid- successional P-J woodlands have in fact declined across the reserve, in part because of loss to fire and in part because of succession into closed-canopy stands. Given this, we suggest that some targeted recovery of open canopy woodland by mechanical thinning be considered both as a means to reduce fuel hazard and associated crown fire risk and to maintain historical fidelity, which remains an important goal for CIRO (W. Keck, CIRO Superintendent, pers. comm.). Thinning could occur around the perimeter of core P-J woodland stands with old-growth characteristics so as to simultaneously protect older stands from fire and to prevent fire spread from woodlands out into steppe vegetation. In particular, areas of the reserve that supported phase III type P-J woodland stands in and around CIRO’s iconic granite monoliths might be appropriately designated as old-growth “reserves”. Reseeding of native steppe vegetation may be necessary in thinned woodland stands where understory is sparse to guard against cheatgrass invasion. The development of a landscape-scale plan for these activities is consistent with recommendations provided by Rodhouse et al. [18], Davies et al. [23], Sheley et al. [24], Weisberg et al. [46], and others (e.g., Shinneman et al. [58]) for maintaining biodiversity and ecological resilience of P-J woodlands and sagebrush steppe in concert. However, we underscore the importance of recognizing the provisional nature of such a plan given the position of CIRO at the range periphery in an era of accelerated change. Because of its position on the regional landscape, CIRO could play a different and more strategic role than it currently does across an increasingly linked network of protected areas in the Great Basin. We hypothesize that CIRO and other protected areas positioned at so-called “biogeographic crossroads” [7] will in fact become increasingly relevant in the future as stepping stones for migration and as harbors of genetically-unique populations pre-adapted to altered climate regimes [4], [6], [7], [59]. But a targeted, careful, adaptive management approach will be critical in these settings both as a way to manage costs as well as to facilitate learning from failures and successes [2], [10]. The change detection approach demonstrated by our study offers a straightforward and accessible means for protected area managers working in woodland range peripheries to gain the necessary insights to craft their own coherent landscape-scale strategies for coping with a changing world.

## Supporting Information

Appendix S1 **A map of fires recorded for CIRO between 1926 and 2005.**
(DOCX)

Appendix S2 **Detailed description of methods used to assess change in woodland cover in City of Rocks National Reserve (CIRO), Idaho.** These methods are written as standard operating procedures for use in future analyses of change at CIRO and in other woodland areas.
(DOCX)
