# Supplementary material for: Woodland Dynamics at the Northern Range Periphery: A Challenge for Protected Area Management in a Changing World
Source: PLoS One. 2013 Jul 29;8(7):e70454. doi: 10.1371/journal.pone.0070454 (PMC3726619; doi:10.1371/journal.pone.0070454)
Supplement: Appendix S1 — A map of fires recorded for CIRO between 1926 and 2005. (DOCX) [file pone.0070454.s001.docx]

Appendix S1: A map of fires recorded for CIRO between 1926 and 2005.


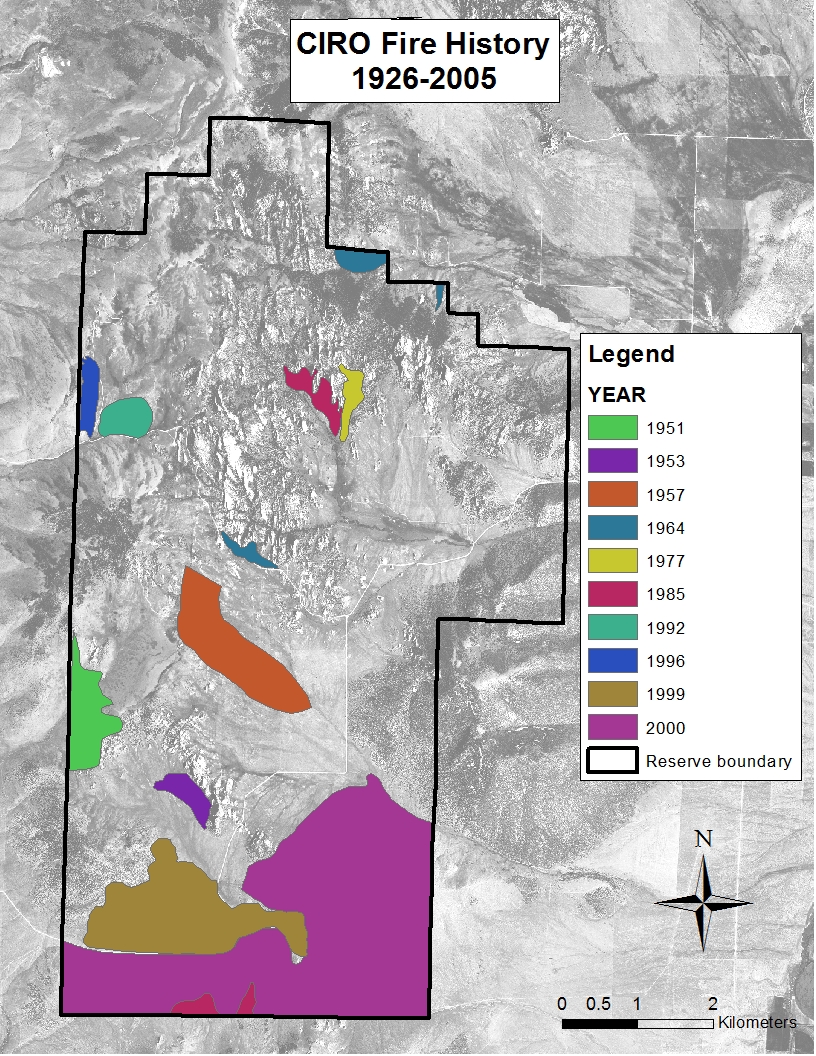


Data Sources:

1) Fire history: Morris LA (2006b) Fire history of the City of Rocks National Reserve from 1926 to 2005. Unpublished report. Logan, UT: Utah State University.

2) Base image: 2009 NAIP image (USDA Farm Service Agency).
