# Supplementary material for: Woodland Dynamics at the Northern Range Periphery: A Challenge for Protected Area Management in a Changing World
Source: PLoS One. 2013 Jul 29;8(7):e70454. doi: 10.1371/journal.pone.0070454 (PMC3726619; doi:10.1371/journal.pone.0070454)
Supplement: Appendix S2 — Detailed description of methods used to assess change in woodland cover in City of Rocks National Reserve (CIRO), Idaho. These methods are written as standard operating procedures for use in future analyses of change at CIRO and in other woodland areas. (DOCX) [file pone.0070454.s002.docx]

Appendix S2: Detailed description of methods used to asses change in woodland cover in City of Rocks National Reserve (CIRO), Idaho. These methods are written as standard operating procedures for use in future analyses of change at CIRO and in other woodland areas.

Contents

Page

[Figures vi](#_Toc342554729)

[Tables vii](#_Toc342554730)

[Introduction 1](#_Toc342554731)

[SOP1: Data Acquisition 2](#_Toc342554732)

[Aerial Photos 2](#_Toc342554733)

[GIS data sets 2](#_Toc342554734)

[SOP2: Study Design 3](#_Toc342554735)

[Overview 3](#_Toc342554736)

[Delineation of study area 3](#_Toc342554737)

[Development of sampling strata 4](#_Toc342554738)

[Elevation 4](#_Toc342554739)

[Solar Radiation 5](#_Toc342554740)

[Vegetation Type and Density 6](#_Toc342554741)

[Combining Stratification Layers 9](#_Toc342554742)

[Clipping stratification layers 12](#_Toc342554743)

[SOP3: Reference Data Collection 13](#_Toc342554744)

[SOP4: Data Analysis 19](#_Toc342554745)

[Summary: 19](#_Toc342554746)

[Variation in rates of change: 19](#_Toc342554747)

[Literature Cited 24](#_Toc342554748)

Figures

Page

[**Figure 1**. Map of the study area (overlaid on a 2009 aerial image) based on the extent of a previous vegetation map (Erixson and Cogan 2011), and the extents of the 1950, 1990, and 2009 aerial photo data sets 4](#_Toc342554777)

[**Figure 2**. Sample plot for aerial photo interpretation. Each plot is a 100 m x 100 m square containing 10 randomly distributed sub-sample points. 15](#_Toc342554778)

[**Figure 3**. Repeat photo examples of transitional phases of woodland succession, Shoshone Mountains, Nevada (From Miller et al. 2008). 17](#_Toc342554779)

Tables

Page

[**Table 1.** Software recommendations for SOPs. 1](#_Toc342554786)

[**Table 2**. Aerial photo specifications. 2](#_Toc342554787)

[**Table 3**. Overview and technical specifications of ancillary GIS data. 2](#_Toc342554788)

[**Table 4**. Vegetation reclassification scheme. 7](#_Toc342554789)

[**Table 5**. Final stratification layer 10](#_Toc342554790)

[**Table 6**. Hierarchical vegetation classification scheme for aerial photo interpretation. 18](#_Toc342554791)

Introduction

This set of Standard Operating Procedures (SOP) accompanies, “Woodland dynamics at the northern range periphery: A challenge for protected area management in a changing world” (Powell et al. *in press*). It is intended to illustrate the key steps for establishing a sample of aerial photo time-series plots to estimate vegetation trends. All data and associated procedures and publications are archived on the National Park Service’s Integrated Resource Management Applications (IRMA) Portal at: <https://irma.nps.gov/App/Reference/Profile/2195678>.

The goal of these SOPs is to establish a consistent methodology that will contribute to improving knowledge of recent dynamics of pinyon-juniper and sagebrush communities to aid in management of this system. Park managers in particular need information on how rates of change vary among biophysical settings, mechanisms underlying past changes, effective methods for monitoring future change, and the potential outcomes of alternative management strategies (National Park Service 2005).

Specific goals of these methods, as demonstrated at City of Rocks National Reserve (CIRO) are as follows:

1. To quantify the rate of change in woody encroachment and densification from 1950-2009 across CIRO based on aerial photographs.

2. To evaluate how rates of vegetation change over this time varied with elevation, solar radiation, and current vegetation type and density.

3. To estimate the spatial extent of changes in conifer, sagebrush steppe and grassland communities at ecotonal boundaries across CIRO.

There are four separate SOPs contained within this document: data acquisition, study design, reference data collection, and data analysis. The following table shows the software (and version) that was used to create these methods (Table 1).

**Table 1.** Software recommendations for SOPs.

| **Software** | **Version** |
| --- | --- |
| ESRI ArcMap | 10.0 |
| Geospatial Modelling Environment | 0.6.0.0 |
| R | 2.14.1 |

Note: Any use of trade, firm, or product names is for descriptive purposes only and does not imply endorsement by the U.S. Government.

SOP1: Data Acquisition

A wide variety of geospatial data sets were used in the development of this SOP, from digital aerial photos to polygon and raster GIS data layers.

Aerial Photos

The aerial photos that were utilized in this study were provided to us in digital format as scanned and geometrically corrected images. Imagery was compiled by the National Park Service Upper Columbia Basin Network (UCBN) from a variety of sources including the National Agricultural Imagery Program (NAIP). We selected three dates of aerial photos for analysis: 1950, 1990, and 2009 (Table 2).

**Table 2**. Aerial photo specifications.

| **Type of Image** | **Year** | **Format** | **Resolution** | **Spatial Extent** | **Source** |
| --- | --- | --- | --- | --- | --- |
| Scanned Aerial Photos | 1950 | B&W | 1 m | CIRO + 1-2 km buffer | UCBN |
| Scanned Aerial Photos | 1990 | Color | 1 m | CIRO + 1-2 km buffer | UCBN |
| NAIP | 2009 | Color | 1 m | CIRO + 5-10 km buffer | USDA |

GIS data sets

In addition to the aerial photos, a wide variety of ancillary GIS data were used to develop this SOP, including raster and polygon GIS data (Table 3). These data were developed from a variety of sources including the USGS, BLM, and NPS and compiled by the UCBN.

**Table 3**. Overview and technical specifications of ancillary GIS data.

| **Data Set** | **Name** | **Format** | **Spatial Extent** | **Source** |
| --- | --- | --- | --- | --- |
| Park Boundary | CIRO_boundary | Polygon shapefile | CIRO | UCBN |
| 10 m Digital Elevation Model (DEM) | ned_10m | GRID | CIRO + 1-2 km buffer | USGS -National Elevation Dataset |
| Fire History | 1.CIRO_FireHistoryreport_polys_1926_2005  2.CIRO_BLM_FirePerimeter_History | Polygon shapefile  Polygon shapefile | CIRO  CIRO + BLM | BLM  BLM |
| Vegetation/  Cover type | draft_ciro_veg_layer | Polygon shapefile | CIRO + 2-5 km buffer | Erixson and Cogan 2011 |

SOP2: Study Design

Overview

Following the methods of Powell and Hansen (2007), we sampled vegetation cover on aerial photographs in plots distributed across key biophysical gradients of the study area. We stratified the study area based upon gradients of vegetation type and density, elevation, and solar radiation, and then generated a stratified random sample of 340 sample plots. We overlaid each 100 m x 100 m sample plot on digital aerial photos from 1950, 1990, and 2009 in a GIS. For each time period, we quantified the percent composition of evergreen, herbaceous/shrub, and deciduous vegetation, as well as the transitional phase of woodland succession (Miller et al. 2008) and recent disturbance events such as fire, insects, and harvest.

The key objective of the study design is to obtain an ample number of reference plots that are distributed across key study area gradients. One important consideration in the design of this study is the size and biophysical variability of the study area. In Powell and Hansen (2007), the study area under consideration was the much larger and more variable Greater Yellowstone Ecosystem, which, therefore, necessitated the establishment of a number of sampling transects distributed across the study area to capture the variability with reasonable personnel effort. For the purposes of this study at CIRO, we determined that due to the relatively small study area (14,407 acres) with modest biophysical gradients (e.g. elevation ranges from 1,646 – 2,706 m), it was equally practical to treat the entire study area as one sampling transect. Therefore, we devised a stratified random sample of the entirety of CIRO.

Delineation of study area

The first step in identification of the actual study area was to determine the geographic extent of the aerial photos and GIS data layers. The goal of this assessment was to determine the minimum overlapping area of the geospatial data layers (Figure 1).

- Determine the spatial overlap and extent of the available aerial photos:
  - 1950 photos: extent covers virtually all of CIRO (with some missing gaps), including some area beyond the boundary (~1-2 km buffer).
  - 1990 photos: extent covers all of CIRO and includes some area beyond the boundary (~1-2 km buffer).
  - 2009 photos: extent covers all of CIRO and includes a substantial buffer beyond boundary (~5-10 km).
- Determine the spatial overlap and extent of the GIS data layers:
  - Vegetation/Cover Type: extent covers all of CIRO and includes some area beyond the boundary (~2-5 km buffer).
  - DEM (elevation/solar radiation): no limit to the available extent.
- **Conclusion**: Based upon the extent and overlap of the aerial photos and GIS data layers, the most practical study area consists of the entirety of CIRO with a small buffer around the perimeter (~1-4 km).

**
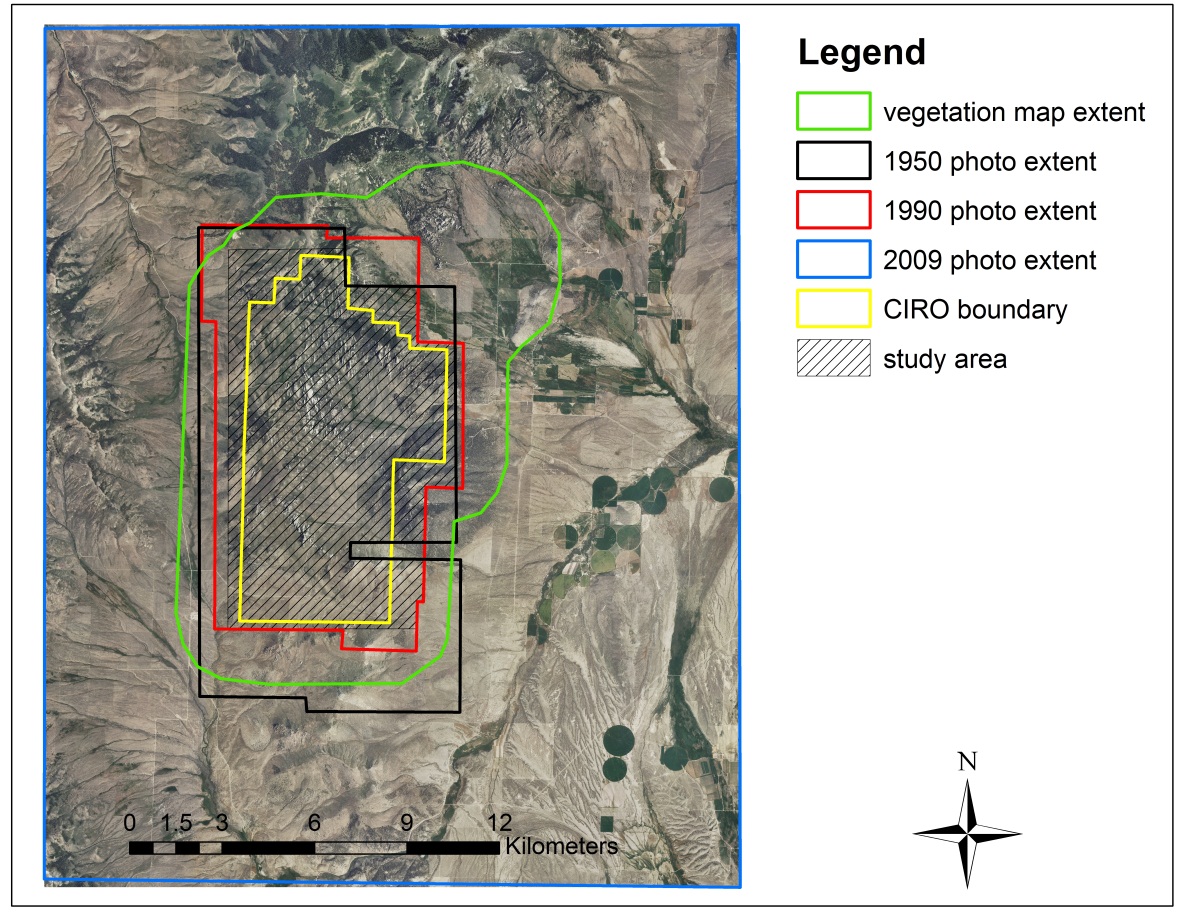
Figure 1**. Map of the study area (overlaid on a 2009 aerial image) based on the extent of a previous vegetation map (Erixson and Cogan 2011), and the extents of the 1950, 1990, and 2009 aerial photo data sets

Development of sampling strata

The objective in the development of sampling strata was to define a set of biophysical variables upon which to base the stratified random sampling. Vegetation type and density, elevation, and solar radiation were selected as the key variables to represent the biophysical variability at CIRO. For the purposes of developing a stratified random sample based on these biophysical factors, it was necessary to create a simplified categorical classification for each of the data layers. The methods for each of the data layers are as follows:

Elevation

Objective: Create a categorical three class elevation layer.

**Step 1**: Project DEM into UTM coordinates

- In ArcMap, add DEM layer to viewer.
- Open ArcToolbox: Select Data Management Tools 🡪Projections and Transformations🡪Raster🡪Project Raster
  - Project Raster dialogue box opens:
    - Input Raster: ned_10m
    - Output Raster: ned_10m_utm
    - Output Coordinate System:
      - Open Spatial Reference Properties Dialogue:
        - Click “Select” button:
        - Choose “Projected Coordinate System”
        - Choose UTM
        - Choose NAD 1983
        - Choose NAD 1983 UTM Zone 12N.prj
    - Accept optional defaults for “Geographic Transformation” and “Output Cell Size”
    - Select “Bilinear” for “Resampling Technique”
    - Click “OK”

**Step 2**: Clip DEM to study area boundary

- Add boundary layer (CIRO_boundary) to viewer
- Open ArcToolbox: Select Data Management Tools 🡪Raster🡪Raster Processing🡪Clip
  - Clip dialogue box opens:
    - Input Raster: ned_10m_utm
    - Output Extent: CIRO_boundary
    - Output Raster Dataset: ned_10m_CIRO
    - Click “OK”

**Step 3**: Reclassify DEM into three classes using Jenks Natural Breaks:

- - - - Open ArcToolbox: Select Spatial Analyst Tools 🡪Reclass🡪Reclassify
  - Reclassify dialogue box opens:
    - Input Raster: ned_10m_CIRO
    - Reclass field: Value
    - Click “Classify” button
    - Select Method: Natural Breaks (Jenks)
    - Choose 3 classes:

1: low elevation: 1672m-1947m

2: mid elevation: 1947m-2177m

3: high elevation: 2177m-2691m

- - - Output raster: elev_3class
    - Click “OK”

Solar Radiation

Objective: Create a categorical three class solar radiation layer from the 10m DEM (ned_10m)

**Step 1**: Create solar radiation surface:

- Derive solar radiation surface from DEM in ArcMap
- Open ArcToolbox: Select Spatial Analyst Tools 🡪 Solar Radiation 🡪 Area Solar Radiation
  - Area Solar Radiation dialogue box opens:
    - Input raster: ned_10m_CIRO
    - Output raster: rad_10m_CIRO
    - Latitude: default - calculated internally from DEM
    - Sky size: default – 200
    - Time configuration: whole year with monthly interval
    - Date/time settings: year=2011
    - Day interval: default – 14
    - Hour interval: 1

**Step 2:** Reclassify solar radiation layer into three classes using Jenks Natural Breaks:

- Open ArcToolbox: Select Spatial Analyst Tools 🡪Reclass🡪Reclassify
  - - - Reclassify dialogue box opens:
    - Input Raster: rad_10m_CIRO
    - Reclass field: Value
    - Click “Classify” button
    - Select Method: Natural Breaks (Jenks)
    - Choose 3 classes

1: low radiation

2: mid radiation

3: high radiation

- Output raster: rad_3class
- Click “OK”

Vegetation Type and Density

Objective: Create a simplified vegetation type/density layer from the 2011 hierarchical CIRO vegetation classification (draft_ciro_veg_layer.shp)

- Original map has 45 classes. Goal: Reclassify into 5 broad types:
  - Evergreen forest and woodland
  - Herbaceous
  - Shrubland
  - Deciduous forest
  - Other (rock, road, water, etc…)

**Step 1:** Convert polygon shapefile layer (draft_ciro_veg_layer.shp) to grid format using the VEG_CODE attribute as the value field.

- Open ArcToolbox: Select Conversion Tools🡪To Raster🡪Feature to Raster
  - Feature to Raster dialogue box opens:
    - Input features: draft_ciro_veg_layer.shp
    - Field: VEG_CODE
    - Output raster: ciro_veg_code
    - Output cell size: 30
    - Click “OK”

**Step 2:** Clip raster layer to study area boundary

- Open ArcToolbox: Select Data Management Tools 🡪Raster🡪Raster Processing🡪Clip
  - Clip dialogue box opens:
    - Input Raster: ciro_veg_code
    - Output Extent: CIRO_boundary
    - Output Raster Dataset: ciro_veg_clip
    - Click “OK”

**Step 3:** Reclassify vegetation layer into five classes:

- Open ArcToolbox: Select Spatial Analyst Tools 🡪Reclass🡪Reclassify
  - - - Reclassify dialogue box opens:
    - Input raster: ciro_veg_clip
    - Reclass field: Value
    - Reclass according to Table 4 below.
    - Output raster: ciro_5class
    - Click “Classify” button
- New five class vegetation codes:
  - - - 1. Evergreen forests and woodland
        2. Herbaceous
        3. Shrubland
        4. Deciduous forest
        5. Other (e.g. rock, agriculture, roads, water)

**Table 4**. Vegetation reclassification scheme.

| **Old VALUE** | **New VALUE** | **VEG_CODE** | **MAP_DESC** |
| --- | --- | --- | --- |
| 1 | **1** | **PSME** | Pseudotsuga menziesii/Acer glabrum forest |
| **2** | **3** | **ARTRV/HERB** | Artemisia tridentata ssp. Vaseyana/Mixed herbaceous shrubland complex |
| **3** | **1** | **PJ/SAGE** | Pinus monophylla woodland |
| **4** | **1** | **CELE** | Cercocarpus ledifolius/Symphoricarpos oreophilus woodland |
| **5** | **4** | **POTR-JUSC** | Populus tremuloides – Juniperus scopulorum woodland |
| **6** | **1** | **PJ/CELE** | Pinus monophylla – (Juniperus osteosperma)/Cercocarpus ledifolius woodland |
| **7** | **3** | **ARTRV** | Artemisia tridentata ssp. Vaseyana/ Symphoricarpos oreophilus shrubland |
| **8** | **5** | **ROCK** | bare rock/sand/other bare ground |
| **9** | **1** | **PJ/SPRS** | Pinus monophylla – Juniperus spp./Sparse understory woodland complex |
| **10** | **2** | **POPR** | Poa pratensis – Juncus balticus semi-natural herbaceous vegetation |
| **11** | **4** | **POTR/MESC** | Populus tremuloides/mesic forest |
| **12** | **5** | **ROAD** | transportation |
| **13** | **4** | **POTR/GRASS** | Populus tremuloides/dry herbaceous forest |
| **14** | **4** | **POAL** | Populus alba – Juniperus scopulorum/Poa pratensis |
| **15** | **2** | **BRTE** | Bromus tectorum semi-natural herbaceous alliance |
| **16** | **3** | **ARTR-PUTR** | Artemisia tridentate – Purshia tridentate shrubland complex |
| **17** | **5** | **CANL** | canal/ditch |
| **18** | **3** | **ARNO** | Artemisia nova/Pseudoroegneria spicata shrubland |
| **19** | **3** | **CEVE** | Ceanothus velutinus shrubland |
| **20** | **3** | **ARAR** | Artemisia arbuscula shrubland alliance |
| **21** | **2** | **CANE** | Carex nebrascensis herbaceous vegetation |
| **22** | **1** | **ABLA** | Abies lasiocarpa/Sparse woodland |
| **23** | **3** | **PUTR** | Purshia tridentate/ Pseudoregneria spicata shrub herbaceous vegetation |
| **24** | **4** | **SABO** | Salix boothi/mesic forbs shrubland |
| **25** | **3** | **SYOR** | Symphoricarpos oreophilus shrubland |
| **26** | **5** | **FILD** | planted/cultivated |
| **27** | **3** | **PRVI** | Prunus virginiana – (Prunus Americana) shrubland |
| **28** | **5** | **MINE** | quarries/strip mines/gravel pits |
| **29** | **5** | **STRM** | stream/river |
| **30** | **2** | **POBU** | Poa bulbosa herbaceous vegetation |
| **31** | **5** | **TRANS** | transitional |
| **32** | **5** | **POND** | lake/pond |
| **33** | **1** | **PIFL** | Pinus flexilis woodland alliance |
| **34** | **5** | **NPS** | NPS facilities |
| **35** | **5** | **AGRI** | Agricultural business |
| **36** | **3** | **ARTRT** | Artemisia tridentata ssp. Tridentate shrubland alliance |
| **37** | **3** | **ARTR/BRTE** | Artemisia tridentata ssp. Vaseyana/mixed herbaceous shrubland complex |
| **38** | **3** | **CHVI** | Chrysothamnus vicidiflorus shrubland alliance |
| **39** | **1** | **PICO** | Pinus contorta woodland alliance |
| **40** | **2** | **PSSP** | Pseudoregneria spicata herbaceous alliance |
| **41** | **1** | **ALIN** | Alnus incana – Juniperus scopulorum shrubland |
| **42** | **2** | **AGDE** | Agropyron desertorum herbaceous vegetation |
| **43** | **3** | **CHNA** | Chrysothamnus nauseosus shrubland alliance |
| **44** | **5** | **RESD** | residential |
| **45** | **2** | **ELLA** | Elymus lanceolatus herbaceous vegetation |

**Step 4:** Use vegetation density attribute to further stratify vegetation classes:

- Convert polygon shapefile layer (draft_ciro_veg_layer.shp) to grid format using the DENS_MOD attribute as the value field.
- Open ArcToolbox: Select Conversion Tools🡪To Raster🡪Feature to Raster
  - Feature to Raster dialogue box opens:
    - Input features: draft_ciro_veg_layer.shp
    - Field: DENS_MOD
    - Output raster: ciro_density
    - Output cell size: 30
    - Click “OK”
- Clip raster layer to study area boundary
- Open ArcToolbox: Select Data Management Tools 🡪Raster🡪Raster Processing🡪Clip
  - Clip dialogue box opens:
    - Input Raster: ciro_density
    - Output Extent: CIRO_boundary
    - Output Raster Dataset: ciro_den_clip
    - Click “OK”
    - ciro_den_clip values:
      - 25-60%
      - 10-25%
      - >60%
      - NA
- Reclassify density layer into three classes (<60%; >60%; NA)
- Open ArcToolbox: Select Spatial Analyst Tools 🡪Reclass🡪Reclassify
  - Reclassify dialogue box opens:
    - Input Raster: ciro_den_clip
    - Output raster: ciro_den_2cl
    - Reclass field: Value
- Old Value: New Value
- 1:1
- 2:1
- 3:2
- 4:3
- Click “OK”
- ciro_den_2cl values:

1: <60%

2: >60%

3: NA

**Step 5:** Combine 5 class vegetation layer with 3 class density layer:

- Open ArcToolbox: Select Spatial Analyst Tools 🡪Map Algebra🡪Raster Calculator
- Raster Calculator dialogue box opens:
- In open box, input the following command (note full layer name containing pathway in available list):
- Combine ([ciro_5class, ciro_den_2cl])
- Output raster: ciro_veg_den
- Click “OK”

**Step 6:** Reclassify vegetation/density layer to remove non-vegetation classes:

- Open ArcToolbox: Select Spatial Analyst Tools 🡪Reclass🡪Reclassify
  - Reclassify dialogue box opens:
    - - - Input Raster: ciro_veg_den
        - Output raster: ciro_veg_den2
        - Reclass field: Value
- Old Value: New Value
- 1:1
- 2:8
- 3:3
- 4:NoData
- 5:5
- 6:6
- 7:2
- 8:7
- 9:4
- Click “OK”
- ciro_veg_den2 values:

Low density shrubland

High density shrubland

Low density herbaceous

High density herbaceous

Low density evergreen forest

High density evergreen forest

Low density deciduous forest

High density deciduous forest

Combining Stratification Layers

Objective: Combine elevation, solar radiation, and vegetation grids for 72 possible classes (68 realized) (Table 5).

- - - - - 8 vegetation classes
        - 3 elevation classes
        - 3 solar radiation classes
        - 72 possible combinations

**Step 1:** Open ArcMap Command Line by clicking on Window tab.

- Open ArcToolbox: Select Spatial Analyst Tools 🡪Map Algebra🡪Raster Calculator
- Raster Calculator dialogue box opens:
- In open box, input the following command (note full layer name containing pathway in available list):
- Combine ([ciro_veg_den2, elev_3class, rad_3class])
- Output raster: vg_den_el_rad
- Click “OK”

**Table 5**. Final stratification layer: values for vg_den_el_rad

| **VALUE** | **COUNT** | **Vegetation/Density** | **Elevation** | **Radiation** |
| --- | --- | --- | --- | --- |
| 1 | 6356 | low density shrubland | high elevation | high radiation |
| 2 | 2834 | low density shrubland | high elevation | mid radiation |
| 3 | 3800 | low density shrubland | high elevation | low radiation |
| 4 | 421 | high density deciduous | high elevation | high radiation |
| 5 | 154 | low density herbaceous | high elevation | high radiation |
| 6 | 1431 | low density coniferous | high elevation | low radiation |
| 7 | 426 | high density deciduous | high elevation | low radiation |
| 8 | 1782 | high density coniferous | high elevation | low radiation |
| 9 | 176 | high density shrubland | high elevation | low radiation |
| 10 | 4194 | low density shrubland | mid elevation | low radiation |
| 11 | 3349 | high density coniferous | mid elevation | low radiation |
| 12 | 9346 | low density shrubland | mid elevation | mid radiation |
| 13 | 1109 | low density herbaceous | mid elevation | mid radiation |
| 14 | 19448 | low density shrubland | low elevation | mid radiation |
| 15 | 6541 | low density shrubland | low elevation | low radiation |
| 16 | 5929 | low density coniferous | low elevation | mid radiation |
| 17 | 3690 | low density coniferous | low elevation | low radiation |
| 18 | 147 | low density deciduous | low elevation | mid radiation |
| 19 | 191 | low density deciduous | low elevation | high radiation |
| 20 | 23178 | low density shrubland | low elevation | high radiation |
| 21 | 9000 | low density coniferous | low elevation | high radiation |
| 22 | 892 | low density coniferous | high elevation | mid radiation |
| 23 | 1601 | low density coniferous | high elevation | high radiation |
| 24 | 10189 | low density shrubland | mid elevation | high radiation |
| 25 | 868 | low density herbaceous | mid elevation | low radiation |
| 26 | 191 | low density herbaceous | high elevation | low radiation |
| 27 | 2989 | high density coniferous | mid elevation | mid radiation |
| 28 | 66 | low density deciduous | low elevation | low radiation |
| 29 | 4627 | low density coniferous | mid elevation | low radiation |
| 30 | 5788 | low density coniferous | mid elevation | mid radiation |
| 31 | 4335 | low density herbaceous | low elevation | mid radiation |
| 32 | 89 | high density deciduous | low elevation | low radiation |
| 33 | 1904 | high density coniferous | high elevation | high radiation |
| 34 | 98 | low density deciduous | high elevation | low radiation |
| 35 | 7710 | low density coniferous | mid elevation | high radiation |
| 36 | 99 | high density shrubland | high elevation | mid radiation |
| 37 | 104 | high density shrubland | high elevation | high radiation |
| 38 | 677 | high density deciduous | high elevation | mid radiation |
| 39 | 581 | high density deciduous | mid elevation | low radiation |
| 40 | 2814 | low density herbaceous | low elevation | high radiation |
| 41 | 411 | high density deciduous | low elevation | mid radiation |
| 42 | 219 | low density deciduous | high elevation | high radiation |
| 43 | 868 | high density deciduous | mid elevation | high radiation |
| 44 | 1018 | high density deciduous | mid elevation | mid radiation |
| 45 | 1291 | high density coniferous | high elevation | mid radiation |
| 46 | 5601 | low density herbaceous | low elevation | low radiation |
| 47 | 161 | low density deciduous | high elevation | mid radiation |
| 48 | 22 | low density deciduous | mid elevation | low radiation |
| 49 | 355 | low density deciduous | mid elevation | mid radiation |
| 50 | 229 | high density shrubland | mid elevation | high radiation |
| 51 | 3702 | high density coniferous | mid elevation | high radiation |
| 52 | 124 | high density shrubland | mid elevation | mid radiation |
| 53 | 107 | high density shrubland | mid elevation | low radiation |
| 54 | 1755 | high density shrubland | low elevation | mid radiation |
| 55 | 266 | high density shrubland | low elevation | low radiation |
| 56 | 1238 | high density shrubland | low elevation | high radiation |
| 57 | 126 | low density herbaceous | high elevation | mid radiation |
| 58 | 533 | low density deciduous | mid elevation | high radiation |
| 59 | 2766 | high density coniferous | low elevation | mid radiation |
| 60 | 2530 | high density coniferous | low elevation | low radiation |
| 61 | 363 | high density deciduous | low elevation | high radiation |
| 62 | 2088 | high density herbaceous | low elevation | high radiation |
| 63 | 842 | high density herbaceous | low elevation | mid radiation |
| 64 | 975 | high density herbaceous | low elevation | low radiation |
| 65 | 779 | low density herbaceous | mid elevation | high radiation |
| 66 | 4036 | high density coniferous | low elevation | high radiation |
| 67 | 195 | high density herbaceous | mid elevation | high radiation |
| 68 | 78 | high density herbaceous | mid elevation | mid radiation |

Clipping stratification layers

Objective: clip vegetation/density/elevation/radiation grid to extent of aerial photos.

**Step 1**: Create polygon extent layers for 1950, 1990, and 2009 aerial photos.

- Open ArcCatalog
  - Navigate to working directory
  - Go to File🡪New🡪Shapefile
    - Enter a name for extent shapefile
    - Feature type: polygon
    - Click “Edit” button for Spatial Reference
      - Spatial Reference Properties dialogue box opens
        - Click “Select”
        - Projected Coordinate Systems
        - UTM
        - NAD 1983
        - NAD 1983 UTM Zone 12N.prj
  - Add polygon extent shapefile in data viewer
  - Go to Editor🡪Start Editing
    - Select directory containing polygon shapefile extent
    - In Editor tool bar, select Task: Create New Feature
    - Select Sketch Tool icon
    - Manually digitize the boundary/extent of aerial photo layer
  - Go to Editor🡪Stop Editing/Save Edits

**Step 2**: Intersect 1950 and 1990 extent polygons to create single layer

- Open ArcToolbox: Select Analysis Tools 🡪Overlay🡪Intersect
  - Intersect dialogue box opens:
    - Input features: 1950_extent.shp; 1990_extent.shp
    - Output feature class: 1950_1990_extent.shp
    - Accept other defaults
    - Click “OK”

**Step 3:** Clip vg_den_el_rad grid layer with 1950_1990_extent.shp layer

- Open ArcToolbox: Select Data Management Tools🡪Raster🡪Raster Processing🡪Clip
  - Clip dialogue opens
    - Input Raster: vg_den_el_rad
    - Output Extent: 1950_1990_extent.shp
    - Output: strat_extent
    - Check box “Use Input Features for Clipping Geometry”

SOP3: Reference Data Collection

The reference data collection methodology is based on aerial photo interpretation. We developed a large sample of aerial photo interpretation plots stratified by vegetation type and density, elevation, and solar radiation. Each of the 340 sample plots was a 100 m x 100 m square overlaid on the digital aerial photos from three time periods. Within each of the sample plots, we randomly generated 10 sample points to guide interpretation of percent vegetation cover. The aerial photo interpretation was organized around a hierarchical vegetation classification scheme (Table 6). This particular classification scheme represents the most detailed levels of classification that could be accurately interpreted.

The first step in the reference data collection methods was to generate a stratified random sample of aerial photo plots based upon the combined vegetation/elevation/solar radiation map.

**Step 1:** Determine appropriate sample size (# plots per stratum):

- We devised a stratified random sample of 340 total plots, equating to approximately 5 plots per stratum.

**Step 2:** Generate random reference points.

- Open ArcToolbox: Select Conversion Tools > From Raster > Raster to Polygon
  - Raster to Polygon dialogue box opens:
    - Input raster: strat_extent
    - Field: value
    - Output Raster: strat_extent_shapefile.shp
    - Uncheck simplify polygons box
- Click “OK”
- Open ArcToolbox: Data Management Tools > Generalization > Dissolve
  - Dissolve dialogue box opens:
    - Input features: strat_extent_shapefile.shp
    - Output Features: strat_extent_dissolve.shp
    - Dissolve Field: GRIDCODE
    - Keep defaults
- Click “OK”
- Open ArcToolbox: Select Data Management Tools>Feature Class>Create Random Points
  - Create Random Points dialogue box opens:
    - Output Location: Specify directory
    - Output Point Feature Class: sample_340_point
    - Constraining Feature Class: strat_extent_dissolve
    - Number of Points: Check “Long” button: 5
    - Click “OK

**Step 3:** Create sample plots from randomly selected points.

- Open Geospatial Modeling Environment from desktop. This is the replacement for Hawth’s Tools in ArcMap 10 (Important note, if this isn’t installed on your computer, go to <http://www.spatialecology.com/gme/gmedownload.htm> for more information about how to download and install):
  - Select Commands Tab
  - Category Filter: Sampling
  - Select genshapes command from list
  - On right side, select Command Builder Tab
    - In: sample_340_point
    - Shape: square
    - Dim: 50 (distance from center point to edge, therefore a 100 m x 100 m square is generated for each sample point)
    - Out: sample_340_square
  - Click “Run”

**Step 4:** Create 10 random points within each plot for interpretation of vegetation cover.

- Open ArcToolbox: Select Data Management Tools 🡪Feature Class🡪Create Random Points
  - Create Random Points dialogue box opens:
    - Output Location: Specify directory
    - Output Point Feature Class: sample_340_square_10pts
    - Constraining Feature Class: sample_340_square.shp
    - Number of Points: Check “Long” button: 10
    - Minimum Allowed Distance: 10 meters
    - Click “OK”

**Step 4:** Populate the attribute table with interpretation fields:

- Add attributes to sample layer attribute table
  - Right click on sample_340_square.shp and select: Open Attribute Table
  - Click on “Table Options” button at top of table
  - Select Add Field; name the field and assign a field type and length (e.g. integer, text)
  - Add the following fields:
    - per_ev_50 (percent evergreen 1950): short integer
    - per_hs_50 (percent herbaceous/shrub 1950): short integer
    - per oth_50 (percent other 1950): short integer
    - per_dec_50 (percent deciduous 1950): short integer
    - type_50 (type 1950): text
    - ss_50 (seral stage 1950): text
    - stat_50 (status 1950): text
    - dist_50 (disturbance 1950): text
    - Add same fields for 1990 and 2009 attributes.
    - Notes (any additional comments): text (length 200)

**Step 5:** Aerial photo interpretation:

Based on the hierarchical vegetation classification scheme for aerial photo interpretation (Table 6), each plot was interpreted for each of the three time periods of aerial photos (1950, 1990, and 2009). An example of a sample plot through time is shown in figure 2.


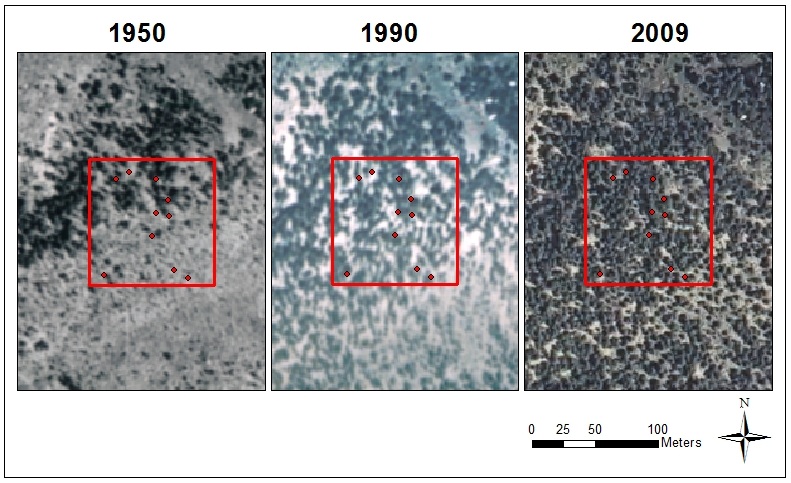


**Figure 2**. Sample plot for aerial photo interpretation. Each plot is a 100 m x 100 m square containing 10 randomly distributed sub-sample points.

The ten randomly distributed points within the plot are used to guide the level 1 “percent composition” of evergreen, deciduous, herbaceous/shrub, or other (rock, water, etc…), recorded between 0 and 100% in 10% increments. Evergreen vegetation in this context includes both needleleaf trees (coniferous: e.g. pinyon pine, juniper, Douglas-fir) and broadleaf trees (e.g. mountain mahogany), while deciduous vegetation strictly refers to broadleaf trees that lose their leaves seasonally (e.g. aspen). This classification distinction was selected because of the difficulty in visually discriminating between evergreen coniferous trees (e.g. pinyon pine) and evergreen broadleaf trees (e.g. mountain mahogany). The herbaceous/shrub category is a physiognomic classification that includes a variety of vegetation types of low stature, including grasses and forbs, as well as evergreen and broadleaf shrubs. Determination of percent composition is based on the point-intercept method (Powell and Hansen 2007) which relies on a tally of intersections between points and vegetation types. For example, in figure 2, it was determined that in 1950, 3 points intersected with evergreen vegetation and 7 points intersected with herbaceous/shrub vegetation. By 1990, 8 of the points intersected with evergreen vegetation and only 2 of the points intersected with herbaceous/shrub vegetation. Finally, by 2009, 9 of the points intersected with evergreen vegetation and 1 of the points intersected with herbaceous/shrub vegetation.

Level 2 “type” is a more detailed assessment of vegetation type for plots containing an evergreen component (needleleaf, broadleaf, or mixed) or an “other” component (water, rocks, etc…). The current vegetation map for CIRO (Erixson and Cogan 2011) was often used to guide the interpretation of level 2 evergreen type. In the example plot in figure 2, the vegetation map indicated that the evergreen component was pinyon pine, therefore, the level 2 “type” was classified as needleleaf throughout the time series.

Level 3 “transitional phase” refers specifically to the successional phase of woodland development described by Miller et al. (2008). Phases 1-3 are specific to plots with an evergreen component, while phase 0 applies to plots that do not contain any measurable tree component (100% herbaceous/shrub). Descriptions and photographic examples from Miller et al. (2008) were often used to guide the interpretation of transitional phase (Figure 3). In the example in figure 2, the plot was classified as phase 1 in 1950, phase 2 in 1990, and phase 3 in 2009.

Level 4 “disturbance” is an interpretation of forest disturbance agents (fire, insects, harvest, other) and year of disturbance if known. Ancillary GIS data for mapped fire events at CIRO were often used to establish the proximate cause and timing of disturbance events for level 4 classification.

It is important to note that aerial photo interpretation is both a technical quantitative exercise as well as a qualitative ecological one. In other words, the 10 randomly generated points within a plot are meant to serve as a guide to interpretation, but by no means should be considered an absolute measure. In some particular instances, the point intercept method will fail to capture a rare cover type that is obvious to the photo interpreter. In that case, the best ecological judgment of the interpreter should trump the absolute quantitative result of the point intercept method.

**Figure 3**. Repeat photo examples of transitional phases of woodland succession, Shoshone Mountains, Nevada (From Miller et al. 2008).

**Table 6**. Hierarchical vegetation classification scheme for aerial photo interpretation.

| **Level 1:**  **% Composition** | **Level 2:**  **Type** | **Level 3:**  **Transitional Phase** | **Level 4: Disturbance** |
| --- | --- | --- | --- |
| % Evergreen | Needleleaf | Phase I  Phase II  Phase III | Fire  Insect  Harvest  Other |
|  | Broadleaf |  |  |
| % Other | Bare  Rock  Agriculture  Water |  | |
| % Herbaceous/  Shrub |  | Phase 0 | |
| % Deciduous |  | | |

Detailed, abbreviated attribute and interpretation codes for aerial photo interpretation:

- **Level 1: % Composition**: 0-100 in 10% increments. Must sum to 100%.
  - % Evergreen: per_ev
  - % Other: per_oth
  - % Herbaceous/Shrub: per_hs
  - % Deciduous: per_dec
- **Level 2: Type**:
  - Needleleaf: ndlf
  - Broadleaf: bdlf
  - mixed needleleaf/broadleaf: mix
  - bare: bare
  - rock: rock
  - agriculture: ag
  - water: water
  - non-natural: nn
- **Level 3: Transitional Phase**
  - Phase 0
  - Phase I
  - Phase II
  - Phase III
- **Level 4: Disturbance**
- Fire: fire
- Insect: ins
- Harvest: har
- other : other

SOP4: Data Analysis

Summary:

To quantify annual rates of woody encroachment, we differenced the percent evergreen composition between time periods and divided by the number of years between observations. For example, if per_ev_50 = 20% and per_ev_09 = 50%, rate of change = (50-20)/59 = 0.51% yr^-1^.

For assessment of variation in rates of change by biophysical gradient, we estimated pairwise differences among all of the biophysical sampling strata using the Bonferonni alpha correction procedure, and report statistically significant differences only when 95% family-wise confidence intervals do not include zero. This approach was chosen to protect against inflated Type I errors greater than the nominal 0.05% rate given the number of pairwise comparisons required.

To estimate the spatial extent of changes in conifer, sagebrush steppe and grassland communities, we reclassified the current CIRO vegetation map (Erixson and Cogan 2011) and the sample plots into four broad classes:

1) sagebrush/shrub/herbaceous/pinyon-juniper

2) mahogany/deciduous

3) other conifer

4) other

To reclassify the sample plots, we assigned the majority vegetation class from the current CIRO vegetation map (Erixson and Cogan 2011) to each sample plot. This reclassification enabled us to focus the analysis specifically on the ecotonal sagebrush and pinyon-juniper communities. Based on the 244 sample plots that were reclassified as sagebrush/shrub/herbaceous/pinyon-juniper, we tallied the number of observations for each time period of the phases of woodland succession based on Miller et al. (2008). All 244 plots, therefore, fell along a continuum from phase 0 of woodland succession (e.g. sagebrush only) to phase 3 (e.g. 100% pinyon-juniper). The proportion of plots in each of the phases was then multiplied by the aerial extent of that class based on the current vegetation map to determine the transitional phase areas for each observation period. For example, based on the current CIRO vegetation map, we determined that the area of sagebrush/shrub/herbaceous/pinyon-juniper was 7,776 ha. From the classification of the phases of woodland succession, we determined that in 2009, 55% of the plots (135 out of 244) were in phase 0 (no measureable tree component). Therefore, we estimated 4,303 ha of phase 0 in 2009. We assumed a constant area of 7,776 ha of sagebrush/shrub/herbaceous/pinyon-juniper between 1950 and 2009 and did not consider transitions that included mahogany/deciduous and other conifer. Phase transitions were then tallied between time periods to determine the relative magnitudes and directions of change. For example, in 1950, there were 120 phase 0 plots, of which 103 plots (86%) remained phase 0 in 1990, 10 plots (8%) transitioned to phase 1, 4 plots (3%) transitioned to phase 2, and 3 plots (3%) transitioned to phase 3.

Variation in rates of change:

The methods to determine statistically significant differences in rates of change among sampling strata were carried out using the following code in R (R Development Core Team 2011).

#############################################################################

#File name: "MultipleComparison.R"

#Purpose: R script for estimating rate of conifer change between

#1950 and 2009 in City of Rocks National Reserve. Example includes analysis #of variance (ANOVA) for difference in mean rates of change between high, #medium, and low elevation strata.

#Note Bonferroni family-wise comparison corrrection factor is used

#to protect against spurious Type I (false-change) error which might arise #when 0.05 alpha is used for a (large) family of among-group tests of #significance.

#See Powell et al. (in press) for additional information.

#NPS Contact: Tom_Rodhouse@nps.gov

*****************************************************************************

#Read example dataset provided with SOP "CIROchange_elev.txt"

setwd();getwd() #set your working directory appropriately where this script #and the dataset are kept.

change<-read.table("CIROchange_elev.txt",header=T)

head(change)

# SAMPLE_340 stratum elevrad elev ever_change rate_change ever_change_50_90

#1 224 4 high_high high 0 0 0

#2 140 4 high_high high 0 0 0

#3 210 4 high_high high 0 0 0

#4 227 4 high_high high 0 0 0

#5 233 42 high_high high 0 0 0

#6 317 42 high_high high 0 0 0

names(change) #use the rate_change for the response per plot

[1] "SAMPLE_340" "stratum" "elevrad"

[4] "elev" "ever_change" "rate_change"

[7] "ever_change_50_90"

#is.factor(change$elev) NOTE: make sure this is true

#Get group means

group.means<-with(change,tapply(rate_change,elev,mean))

high low mid

0.05649718 0.20216459 0.10946328

#get group sample sizes

group.length<-with(change,tapply(rate_change,elev,length))

high low mid

105 83 96

#Let's take a look! Boxplots!!!

with(change,boxplot(rate_change~elev))

#Are these rates of change significantly different from one another? Note

#the rate of change in low elevation strata appears noticeably higher.

fm1=with(change,aov(rate_change~elev))

summary(fm1)

Df Sum Sq Mean Sq F value Pr(>F)

elev 2 0.992 0.4959 10.73 3.24e-05 ***

Residuals 281 12.991 0.0462

---

Signif. codes: 0 ‘***’ 0.001 ‘**’ 0.01 ‘*’ 0.05 ‘.’ 0.1 ‘ ’ 1

#Yes, there seems to be a difference in means between at least 1 pair of #strata.

*****************************************************************************

#Create multiple comparisons functions - run this section all together.

#note this will provide 3 diff't multiple comparisons procedures to choose #from.

#This function written by Jim Robison-Cox

#(http://www.agr.kuleuven.ac.be/vakken/statisticsbyR/ANOVAbyRr/multiplecompJI#MRC.htm).

#See the "multcomp" package (Hothorn et al. 2008) as another source for code.

all.pairs <- function(r)

list(first = rep(1:r,rep(r,r))[lower.tri(diag(r))],

second = rep(1:r, r)[lower.tri(diag(r))])

tukeyCI <- function(fitted, nis, df, MSE, conf.level=.95){

## fitted is a sequence of means

## nis is a corresponding sequence of sample sizes for each mean

## df is the residual df from the ANOVA table

## MSE = mean squared error from the ANOVA table

## conf.level is the family-wise confidence level, defaults to .95

r <- length(fitted)

pairs <- all.pairs(r)

diffs <- fitted[pairs$first] - fitted[pairs$second]

df <- sum(nis) - r

T <- qtukey(conf.level, r, df)/sqrt(2)

hwidths <- T*sqrt(MSE*(1/nis[pairs$first] + 1/nis[pairs$second]))

val <- cbind(diffs - hwidths, diffs, diffs + hwidths)

dimnames(val) <- list(paste("mu",pairs$first," - mu", pairs$second,

sep=""), c("Lower", "Diff","Upper"))

val

}

scheffeCI <- function(fitted, nis, df, MSE, conf.level=.95){

## fitted is a sequence of means

## nis is a corresponding sequence of sample sizes for each mean

## df is the residual df from the ANOVA table

## MSE = mean squared error from the ANOVA table

## conf.level is the family-wise confidence level, defaults to .95

r <- length(fitted)

pairs <- all.pairs(r)

diffs <- fitted[pairs$first] - fitted[pairs$second]

T <- sqrt((r-1)*qf(conf.level,r-1,df))

hwidths <- T*sqrt(MSE*(1/nis[pairs$first] + 1/nis[pairs$second]))

val <- cbind(diffs - hwidths, diffs, diffs + hwidths)

dimnames(val) <- list(paste("mu",pairs$first," - mu", pairs$second,

sep=""), c("Lower", "Diff","Upper"))

val

}

bonferroniCI <- function(fitted, nis, df, MSE, conf.level=.95){

## fitted is a sequence of means

## nis is a corresponding sequence of sample sizes for each mean

## df is the residual df from the ANOVA table

## MSE = mean squared error from the ANOVA table

## conf.level is the family-wise confidence level, defaults to .95

r <- length(fitted)

pairs <- all.pairs(r)

diffs <- fitted[pairs$first] - fitted[pairs$second]

T <- qt(1-(1-conf.level)/(2*r*(r-1)),df)

hwidths <- T*sqrt(MSE*(1/nis[pairs$first] + 1/nis[pairs$second]))

val <- cbind(diffs - hwidths, diffs, diffs + hwidths)

dimnames(val) <- list(paste("mu",pairs$first," - mu", pairs$second,

sep=""), c("Lower", "Diff","Upper"))

val

}

*****************************************************************************

#a few other inputs from the ANOVA

dfMSE=fm1$df.residual;dfMSE #281 see above ANOVA summary

MSE=sum(fm1$residuals^2)/dfMSE;MSE #mean squared error

#There are different types of multi-comparisons, with pros and cons

#Tukey's HSD (Note mu1 is high, mu2 is low, mu3 is mid (this could be fixed

#if you want to reorder the input text file)

tukeyCI(group.means, group.length, dfMSE, MSE, conf=.95)

Lower Diff Upper

mu1 - mu2 -0.22007568 -0.14566742 -0.07125915

mu1 - mu3 -0.12450525 -0.05296610 0.01857304

mu2 - mu3 0.01676879 0.09270131 0.16863384

#Scheffe's correction

scheffeCI(group.means, group.length, dfMSE, MSE, conf=.95)

Lower Diff Upper

mu1 - mu2 -0.22338056 -0.14566742 -0.06795427

mu1 - mu3 -0.12768268 -0.05296610 0.02175048

mu2 - mu3 0.01339622 0.09270131 0.17200641

#bonferroni correction

bonferroniCI(group.means, group.length, dfMSE, MSE, conf=.95)

Lower Diff Upper

mu1 - mu2 -0.229576950 -0.14566742 -0.06175788

mu1 - mu3 -0.133640149 -0.05296610 0.02770795

mu2 - mu3 0.007072892 0.09270131 0.17832973

#######################

#NOTE: with each of these methods we see that lower elevation (mu2) is

#significantly larger than high elevation (mu1), and significantly

#larger than medium elevation (mu3). There is no significant difference

#between high elevation(mu1) and medium elevation (mu2).

#This code can be modified to run multiple comparisons between different

#group(strata) combinations (e.g., "elevrad" - the combination of elevation #and solar radiation groups)

#END SCRIPT

##############################################################################

Literature Cited

Erixson, J., and D. Cogan. 2011. Vegetation inventory project report: City of Rocks National Reserve. Natural Resource Report NPS/UCBN/NRR-2011/450. National Park Service, Fort Collins, Colorado.

Miller, R. F., R. J. Tausch, D. E. McArthur, D. Johnson, and S. C. Sanderson. 2008. Age structure and expansion of pinon-juniper woodlands: a regional perspective in the Intermountain West. Research Paper RMRS-RP-69. Fort Collins, CO, USA: USDA Forest Service, Rocky Mountain Research Station. 15 p.

National Park Service. 2005. City of Rocks National Reserve Fire Management Plan. Prepared by the National Park Service West Region Fire Program and the City of Rocks National Reserve staff with assistance from Hagerman Fossil Beds National Monument staff. Published by the National Park Service, City of Rocks National Reserve.

Powell, S. L. and A. J. Hansen. 2007. Conifer cover increase in the Greater Yellowstone Ecosystem: frequency, rates, and spatial variation. *Ecosystems* 10:204-216.

Powell, S.L., A.J. Hansen, T.J. Rodhouse, L. K. Garrett, J.L. Betancourt, G.H. Dicus, and M.K. Lonneker. In press. Woodland dynamics at the northern range periphery: A challenge for protected area management in a changing world. *PLOS ONE.*

R Development Core Team. 2011. R: A language and environment for statistical computing. R Foundation for Statistical Computing, Vienna, Austria. ISBN 3-900051-07-0, URL: <http://www.R-project.org/>.
